# Efficient class of ratio cum median estimators for estimating the population median

**DOI:** 10.1371/journal.pone.0274690

**Published:** 2023-02-09

**Authors:** Mir Subzar, Showkat Ahmad Lone, Emmanuel J. Ekpenyong, Abdul Salam, Muhammad Aslam, T. A. Raja, Salmeh A. Almutlak

**Affiliations:** 1 Department of Mathematics, (SSBSR), Sharda University, Greater Noida, UP, India; 2 Department of Basic Sciences, College of Science and Theoretical Studies, Saudi Electronic University, Riyadh, Saudi Arabia; 3 Department of Statistics, Michael Okpara University of Agriculture, Umudike, Abia, Nigeria; 4 Department of Epidemiology, College of Public Health & Tropical Medicine, Jazan University, Jazan, Saudi Arabia; 5 Department of Statistic, Faculty of Science, King Abdul-Aziz University, Jeddah, Saudi Arabia; 6 Division of Agricultural Economics & Statistics, FoA, Wadura, SKUAST-Kashmir, Srinagar, India; University of the Punjab, PAKISTAN

## Abstract

In estimation theory, the use of auxiliary information significantly improves precision while estimating population parameters. In this paper, an efficient class of ratio cum median estimators of the population median is suggested using simple random sampling without replacement. The expressions for bias and mean square error of the proposed class are derived theoretically. The condition for the asymptotic optimum estimator is obtained with its bias and mean square error expressions. Under certain realistic conditions, the asymptotic optimum estimator is more proficient, based on analytical and numerical comparisons with some existing estimators that are members of the suggested class of estimators. The superiority of the proposed ratio cum median estimators is shown through real data applications. Such a new proposed estimator will be useful in the future for data analysis and making decisions.

## 1. Introduction

The use of auxiliary information, either at the selection or estimation stage or at both stages, significantly improves precision while estimating population parameters. Sometimes, in survey sampling, the collected data may follow the normal distribution. In that case, we use the mean and ordinary least squares methods to estimate the population parameters, as these methods give accurate and precise results in that situation. However, in most cases, data collected may not follow the normal distribution (for example, salary, consumption, etc.), but may follow some highly skewed distributions. In these situations, using the value of the mean will not provide accurate and precise results, as the mean is too sensitive to outliers. So, one can use another measure of central tendencies, such as the median since it is not sensitive to extreme values or outliers. Researchers usually find it difficult to propose such a new technique in order to obtain a valid inference in such situations. Therefore, in this present study, we tend to develop a family of estimators of the population median, which could be adopted whenever the population distribution is skewed. Hence, we suggest a family of ratio cum median estimators of population median by using a combination of scalars with the known population median of a supplementary variable. Also, the asymptotic optimum estimator (AOE) conditions are obtained under which the suggested estimator is highly efficient and consistent, and compares favourably amongst other estimators of the median.

Consider *R* to be the supplementary variable and *S* to be the response variable of the finite population under study. Let *r*_*i*_ and *s*_*i*_ be the sample variables on each *i*^*th*^ unit drawn from *R*_*i*_ and *S*_*i*_ variables by simple random sampling without replacement (SRSWOR). Let *t*_*r*_(*r*) and *t*_*s*_(*s*) are the marginal densities of r and s respectively; *t*_*r*_(*H*_*r*_) and *t*_*s*_(*H*_*s*_) are the probability density functions of the variables. Moreover, let ${\hat{H}}_{r}$ and ${\hat{H}}_{s}$ be the sample medians; *H*_*r*_ and *H*_*s*_, the population medians of the variables with the correlation coefficient

$$\rho_{c}=4p_{11}(s,r)-1;p_{11}(r,s)=p(S\leq H_{s}\cap R\leq H_{r}).$$

$$\mathrm{Let}\;e_{s}=(({\hat{H}}_{s}-H_{s})/H_{s}),\;\mathrm{and}\;e_{r}=(({\hat{H}}_{r}-H_{r})/H_{r}).$$ (1.1)

Thus,

$$E(e_{r})=E(e_{s})=0,E(e_{s}^{2})=\delta C_{Hs}^{2},E(e_{r}^{2})=\delta C_{Hr}^{2},E(e_{r}e_{s})=\delta \rho_{c}C_{Hs}C_{Hr},$$ (1.2)

where

$$C_{Hs}={\left[H_{s}t_{s}(H_{s})\right]}^{-1},C_{Hr}={\left[H_{r}f_{r}(H_{r})\right]}^{-1},t=m/M,k_{H}=\rho_{c}C_{Hs}/C_{Hr},$$ (1.3)

Different estimators with their properties are recommended by different authors in different situations, taking into consideration the above-mentioned population. [1] recommended the usual unbiased median estimator ${\hat{H}}_{s}$ and is given with the expression of variance as

$$Var({\hat{H}}_{s})=\delta H_{s}^{2}C_{Hs}^{2}.$$ (1.4)

[2] suggested the classical ratio median estimator given as ${\hat{H}}_{R}={\hat{H}}_{s}(H_{r}/{\hat{H}}_{r})$ with bias, $Bias({\hat{H}}_{R})=\delta H_{s}C_{Hr}^{2}(1-k_{H})$ and its mean square error,

$$MSE({\hat{H}}_{R})=\delta H_{s}^{2}[C_{Hs}^{2}+C_{Hr}^{2}(1-2k_{H})]$$ (1.5)

[3, 4] suggested classical product-type median estimator as ${\hat{H}}_{p}={\hat{H}}_{s}({\hat{H}}_{r}/H_{r})$ with bias, $Bias({\hat{H}}_{p})=\delta H_{s}C_{Hr}^{2}k_{H}$ and its mean square error,

$$MSE({\hat{H}}_{p})=\delta H_{s}^{2}[C_{Hs}^{2}+C_{Hr}^{2}(1+2k_{H})]$$ (1.6)

[5] recommended the exponential ratio type median estimators

${\hat{H}}_{\exp R}={\hat{H}}_{s}\exp[\left(H_{r}-{\hat{H}}_{r}\right)/\left(H_{r}+{\hat{H}}_{r}\right)]$. Its bias is expressed as: $Bias({\hat{H}}_{\exp R})=(\delta H_{s}C_{Hr}^{2}(3-4k_{H}))/8$, and its mean square error is

$$MSE({\hat{H}}_{\exp R})=\delta H_{s}^{2}[C_{Hs}^{2}+(C_{Hr}^{2}/4)(1-4k_{h})]$$ (1.7)

Also, [5] recommended the exponential product type median estimator with its bias and mean square error expression as

${\hat{H}}_{\exp P}={\hat{H}}_{s}\exp[\left({\hat{H}}_{r}-H_{r}\right)/\left(H_{r}+{\hat{H}}_{r}\right)]$ with bias $Bias({\hat{H}}_{\exp R})=(\delta H_{s}C_{Hr}^{2}(4k_{H}-1))/8$ and its mean square error

$$MSE({\hat{H}}_{\exp P})=\delta H_{s}^{2}[C_{Hs}^{2}+(C_{Hr}^{2}/4)(1+4k_{H})]$$ (1.8)

[6] Suggested the classical chain ratio type median estimator and is given with its bias and mean square error as ${\hat{H}}_{CR}={\hat{H}}_{y}{\left(H_{r}/{\hat{H}}_{r}\right)}^{2}$ with bias $Bias({\hat{H}}_{CR})=\delta H_{s}C_{Hr}^{2}(1+2k_{H})$ and its Mean square error

$$MSE({\hat{H}}_{CR})=\delta H_{s}^{2}[C_{Hs}^{2}+4C_{Hr}^{2}(1+2k_{H})]$$ (1.9)

For more works on estimators of population median, we refer the readers to [7–9]. [10] Considered the randomized response method in estimating the population ratio based on ranked set sampling. [11, 12] investigated the population median estimation in two-stage ranked set sampling, and multistage ranked set sampling [13]. Suggested an estimator for the population mean using stratified median ranked set sampling [14]. Considered the case of median estimation with the imputation of the missing observations [15]. Suggested ratio estimators of population mean using auxiliary information in simple random sampling and median ranked set sampling [16]. Considered the missing values using ranked set sampling for ratio estimation of the population mean.

Other parts of the paper are organized as follows. In Section 2, the proposed class of ratio estimators for the population median is provided. The bias and mean square error of the proposed class of estimators are given in Section 3. The Asymptotic optimal estimator for the family of these estimators is considered and presented in Section 4. In Section 5, some existing members of the proposed class of estimators are summarized. Efficiency comparisons are presented in Section 6 while empirical investigations are given in Section 7 for real data illustration. The paper is concluded in Section 8 with some suggestions for future works.

## 2. The proposed class of ratio estimators for population median

In this section, we have proposed a ratio estimator for population median as

$$q_{\left(\eta ,\psi \right)}={\hat{H}}_{s}\{2-{\left(\frac{{\hat{H}}_{r}}{H_{r}}\right)}^{\eta }\exp[\frac{\psi ({\hat{H}}_{r}-H_{r})}{\left({\hat{H}}_{r}+H_{r}\right)}]\}.$$ (2.1)

Then, we set the above-proposed estimator’s boundary conditions: if ${\left(\frac{{\hat{H}}_{r}}{H_{r}}\right)}^{\eta }\to 1$, $\{\exp[\frac{\psi ({\hat{H}}_{r}-H_{r})}{\left({\hat{H}}_{r}+H_{r}\right)}]\}\to 1$ and $E({\hat{H}}_{r})=H_{r}$, $E[q_{\left(\eta ,\psi \right)}]\to H_{s}$ making the estimator *q*_(*η*,*ψ*)_ unbiased. This justifies the use of the number 2 in (2.1)

**Theorem 1:** If in *q*_(*η*,*ψ*)_ different scalars are being set, such that 2→*θ* and $u{\left(\frac{{\hat{H}}_{r}}{H_{r}}\right)}^{\eta }\exp[\frac{\psi ({\hat{H}}_{r}-H_{r})}{\left({\hat{H}}_{r}+H_{r}\right)}]$ is used to replace ${\left(\frac{{\hat{H}}_{r}}{H_{r}}\right)}^{\eta }\exp[\frac{\psi ({\hat{H}}_{r}-H_{r})}{\left({\hat{H}}_{r}+H_{r}\right)}]$ in (2.1), the new class of estimators

$$q_{\left(\theta ,u,\eta ,\psi \right)}={\hat{H}}_{s}\{\theta -u{\left(\frac{{\hat{H}}_{r}}{H_{r}}\right)}^{\eta }\exp[\frac{\psi ({\hat{H}}_{r}-H_{r})}{\left({\hat{H}}_{r}+H_{r}\right)}]\}$$ (2.2)

is the adjustment of (2.1) for suitably chosen scalars *θ*,*u*,*η*,*ψ* such that *θ* and *u* satisfy the condition

θ=1+u;−∞<u<∞ (2.3)

## 3. The bias and mean square error of the proposed class of estimators

Let,

$${\hat{H}}_{r}=H_{r}(1+e_{r}),{\hat{H}}_{s}=H_{r}(1+e_{s}),e_{r}=\left({\hat{H}}_{r}-H_{r}\right)/H_{r},e_{s}=\left({\hat{H}}_{s}-H_{s}\right)/H_{s}$$ (3.1)

Therefore, expressing (2.2) in terms of (3.1) results in an approximate expression for the bias and the MSE for the proposed class of estimators. Thus, we have

$$q_{\left(\theta ,u,\eta ,\psi \right)}=H_{s}(1+e_{s})\{\theta -u{\left(\frac{H_{r}(1+e_{r})}{H_{r}}\right)}^{\eta }\exp[\frac{\psi (H_{r}(1+e_{r})-H_{r})}{2H_{r}(1+e_{r}/2)}]\}$$

$$=H_{s}(1+e_{s})\{\theta -u{\left(1+e_{r}\right)}^{\eta }\exp[\frac{\psi H_{r}e_{r}}{2H_{r}(1+e_{r}/2)}]\}$$

$$=H_{s}(1+e_{s})\{\theta -u{\left(1+e_{r}\right)}^{\eta }\exp[\frac{\psi e_{r}}{2}{\left(1+e_{r}/2\right)}^{-1}]\}$$

Assuming that |*e*_*r*_|<1 and expanding (1+*e*_*r*_)^*η*^, $\left[\frac{\psi e_{r}}{2}{\left(1+e_{r}/2\right)}^{-1}\right]$ and (1+*e*_*r*_/2)^−1^, we obtain

$$q_{\left(\theta ,u,\eta ,\psi \right)}=H_{s}\left(1+e_{s}\right)\{\theta -u[1+\eta e_{r}+\frac{\eta (\eta -1)}{2}e_{r}^{2}+\ldots ][1+\frac{\psi e_{r}}{2}{\left(1+e_{r}/2\right)}^{-1}+\frac{\psi^{2}e_{r}^{2}}{8}{\left(1+e_{r}/2\right)}^{-2}]\}$$

$$=H_{s}\left(1+e_{s}\right)\{\theta -u[1+\eta e_{r}+\frac{\eta (\eta -1)}{2}e_{r}^{2}+\ldots ][1+[\frac{\psi e_{r}}{2}(1-\frac{e_{r}}{2}+\frac{e_{r}^{2}}{4}-\ldots )]+\frac{\psi^{2}e_{r}^{2}}{8}(1-e_{r})]\}$$

$$=H_{s}\left(1+e_{s}\right)\{\theta -u[1+\eta e_{r}+\frac{\eta (\eta -1)}{2}e_{r}^{2}+\ldots ][1+\frac{\psi e_{r}}{2}-\frac{\psi e_{r}^{2}}{4}+\frac{\psi^{2}e_{r}^{2}}{8}]\}$$

$$=H_{s}\left(1+e_{s}\right)\{\left(\theta -u)-\frac{ue_{r}}{2}(2\eta +\psi )-\frac{ue_{r}^{2}}{8}(4\eta^{2}-4\eta -2\psi +\psi^{2}+4\eta \psi \right)\}$$

$$=H_{s}\left(1+e_{s}\right)\{(\theta -u)-\frac{u(2\eta +\psi )e_{r}}{2}-\frac{u(2\eta +\psi )(2\eta +\psi -2)e_{r}^{2}}{8}\}$$

By neglecting the terms of *e*_*i*_(*i* = *r or s*) having power greater than two, we have

$$=H_{s}\{(\theta -u)-\frac{u(2\eta +\psi )e_{r}}{2}-\frac{u(2\eta +\psi )(2\eta +\psi -2)e_{r}^{2}}{8}+(\theta -u)e_{s}-\frac{u(2\eta +\psi )e_{r}e_{s}}{2}\}$$

$$=H_{s}\{(\theta -u)-\frac{u(2\eta +\psi )}{2}[e_{r}+\frac{(2\eta +\psi -2)e_{r}^{2}}{4}+e_{r}e_{s}]+(\theta -u)e_{s}\}$$ (3.2)

Therefore,

$$q_{\left(\theta ,u,\eta ,\psi \right)}-H_{s}≅H_{s}\{(\theta -u-1)-\frac{u(2\eta +\psi )}{2}[e_{r}+\frac{(2\eta +\psi -2)e_{r}^{2}}{4}+e_{r}e_{s}]+(\theta -u)e_{s}\}$$ (3.3)

Hence, the bias for the proposed class of ratio estimators *q*_(*θ*,*u*,*η*,*ψ*)_ to the first-degree approximation is obtained. Taking the expectation of (3.3) and using (1.2), the bias is given by

$$Bias[q_{\left(\theta ,u,\eta ,\psi \right)}]=E[q_{\left(\theta ,u,\eta ,\psi \right)}-H_{s}]$$

$$≅(\theta -u-1)H_{s}+\frac{\left(1-t\right)}{m}H_{s}\{-\frac{u(2\eta +\psi )}{2}[\frac{\left(2\eta +\psi -2\right)}{4}C_{Mr}^{2}+\rho_{c}C_{Hs}C_{Hr}]\}$$ (3.4)

Now, by neglecting the terms having powers greater than two in *e*_*i*_(*i* = *r or s*), squaring both sides, taking expectation in (3.3) and substituting (1.2), the MSE to the first-degree approximation is obtained for the recommended class of ratio cum median estimator as

$$MSE[q_{\left(\theta ,u,\eta ,\psi \right)}]=E{\left[q_{\left(\theta ,u,\eta ,\psi \right)}-H_{s}\right]}^{2}$$

$$=E{\left\{H_{s}[(\theta -u-1)-\frac{u(2\eta +\psi )}{2}e_{r}+(\theta -u)e_{s}]\right\}}^{2}$$

$$=E\{H_{s}^{2}[\begin{array}{l} {\left(\theta -u-1\right)}^{2}-\frac{u^{2}e_{r}^{2}{\left(2\eta +\psi \right)}^{2}}{4}+{\left(\theta -u\right)}^{2}e_{s}^{2}+2(\theta -u-1)(\theta -u)e_{s}\\ -(\theta -u-1)(2\eta +\psi )ue_{r}-u(2\eta +\psi )(\theta -u)e_{s}e_{r} \end{array}]\}$$

$$=H_{s}^{2}{\left(\theta -u-1\right)}^{2}+\frac{\left(1-t\right)}{m}H_{s}^{2}[{\left(\theta -u\right)}^{2}C_{Hs}^{2}+\frac{u^{2}{\left(2\eta +\psi \right)}^{2}}{4}C_{Hr}^{2}-u(2\eta +\psi )(\theta -u)\rho_{c}C_{Hs}C_{Hr}]$$

$$=H_{s}^{2}{\left(\theta -u-1\right)}^{2}+\frac{\left(1-t\right)}{m}H_{s}^{2}[{\left(\theta -u\right)}^{2}C_{Hs}^{2}+\frac{\left(2\eta +\psi \right)}{4}C_{Hr}^{2}[u^{2}(2\eta +\psi )-4k_{H}u(\theta -u)]]$$ (3.5)

## 4. The optimal condition for the proposed class of estimators

To investigate the optimal conditions for the proposed class of estimators, let $\frac{\partial MSE(q_{\left(\theta ,u,\eta ,\psi \right)})}{\partial \eta }=0$ so that,

$$u^{2}C_{Hs}^{2}(2\eta +\psi )=2k_{H}u(\theta -u)C_{Hr}^{2}\Rightarrow (2\eta +\psi )=\frac{2k_{H}(\theta -u)}{u}=\frac{2k_{H}}{u},$$ (4.1)

Hence, substituting (4.1) into (3.5), we obtain

$$MSE[q_{\left(\theta ,u,\eta ,\psi \right)}]=H_{s}^{2}{\left(\theta -u-1\right)}^{2}+\frac{\left(1-t\right)}{m}H_{s}^{2}[\begin{array}{l} {\left(\theta -u\right)}^{2}C_{Hs}^{2}+\frac{2k_{H}(\theta -u)}{4u}C_{Hr}^{2}\times \\ \left[\frac{2k_{H}u^{2}(\theta -u)}{u}-4k_{H}u(\theta -u)\right] \end{array}]$$

$$=H_{s}^{2}{\left(\theta -u-1\right)}^{2}+\frac{\left(1-t\right)}{m}H_{s}^{2}[{\left(\theta -u\right)}^{2}C_{Hs}^{2}+\frac{k_{H}(\theta -u)}{2u}C_{Hr}^{2}[-2k_{H}u(\theta -u)]]$$

$$=H_{s}^{2}{\left(\theta -u-1\right)}^{2}+\frac{\left(1-t\right)}{m}H_{s}^{2}[{\left(\theta -u\right)}^{2}C_{Hs}^{2}-{\left(\theta -u\right)}^{2}k_{H}^{2}C_{Hr}^{2}]$$

$$=H_{s}^{2}{\left(\theta -u-1\right)}^{2}+\frac{\left(1-t\right)}{m}H_{s}^{2}{\left(\theta -u\right)}^{2}[C_{Hs}^{2}-k_{H}^{2}C_{Hr}^{2}]$$

$$=H_{s}^{2}{\left(\theta -u-1\right)}^{2}+\frac{\left(1-t\right)}{m}H_{s}^{2}{\left(\theta -u\right)}^{2}C_{Hs}^{2}[1-\rho_{c}^{2}]$$ (4.2)

Finally, substituting (3.3) in (4.2), we obtain the asymptotic MSE for the proposed class of estimators *z*_(*θ*,*u*,*η*,*ψ*)_ as

$$MSE[q_{\left(\theta ,u,\eta ,\psi \right)}]=\frac{\left(1-t\right)}{m}H_{s}^{2}C_{Hs}^{2}(1-\rho_{c}^{2})=\delta H_{s}^{2}C_{Hs}^{2}(1-\rho_{c}^{2}).$$ (4.3)

## 5. Some existing members of the proposed class of estimators

In this section, we will show how some existing estimators are members of the suggested class of ratio cum median estimator when certain suitable values are assumed by scalars *θ*,*u*,*η*,*ψ*, with their exact expressions of biases and mean square errors. Table 1 provides defined values of *θ*,*u*,*η*,*ψ* that will produce the existing members of the suggested class of estimators mentioned in this study.

**Table 1:** Some existing members of the proposed class of estimators.

*θ*	*u*	*η*	*ψ*	Estimators
1	0	*η*	*ψ*	${\hat{H}}_{s}$ Unbiased Usual Median estimator
0	-1	-1	0	${\hat{H}}_{R}={\hat{H}}_{s}(H_{r}/{\hat{H}}_{r})$ Classical ratio median estimator
0	-1	1	0	${\hat{H}}_{P}={\hat{H}}_{s}({\hat{H}}_{r}/H_{r})$ Product type median estimator
0	-1	0	-1	${\hat{H}}_{\exp R}={\hat{H}}_{s}\exp[\left(H_{r}-{\hat{H}}_{r}\right)/\left(H_{r}+{\hat{H}}_{r}\right)]$
				Bahl and Tuteja exponential ratio median estimator
0	-1	0	1	${\hat{H}}_{\exp R}={\hat{H}}_{s}\exp[\left({\hat{H}}_{r}-H_{r}\right)/\left(H_{r}+{\hat{H}}_{r}\right)]$
				Bahl and Tuteja exponential product median estimator
0	-1	2	0	${\hat{H}}_{CR}={\hat{H}}_{s}{\left(H_{r}/{\hat{H}}_{r}\right)}^{2}$ Chain ratio median estimator

## 6. Efficiency comparisons

Here, theoretical comparisons have been made between the suggested ratio-cum-median estimator at its optimal condition with the rest of the estimators, which are members of this recommended estimator, when *θ*,*u*,*η*,*ψ*, assume certain unique values. To make a comparison, we take the final expression of the mean square error of the proposed estimator at its optimal condition and the MSE expressions of the estimators, which are members of this estimator.

**(a) Comparison of** *MSE*_*opt*_[*q*_(*θ*,*u*,*η*,*ψ*)_] **with sample median estimator ${\hat{H}}_{s}$**

Comparing (1.4) and (4.3), (*optimum*)*q*_(*θ*,*u*,*η*,*ψ*)_ will be more efficient than ${\hat{H}}_{s}$ if

That is

$\Rightarrow \delta H_{s}^{2}C_{Hs}^{2}\rho_{c}^{2}>0$, which is always true.

**(b) Comparison of** *MSE*_*opt*_[*q*_(*θ*,*u*,*η*,*ψ*)_] **with classical ratio median estimator ${\hat{H}}_{R}$**

Comparing (1.5) and (4.3), (*optimum*)*q*_(*θ*,*u*,*η*,*ψ*)_ will be more efficient than ${\hat{H}}_{R}$ if

$$\delta H_{s}^{2}[C_{Hs}^{2}+C_{Hr}^{2}(1-2k_{H})]-\delta H_{s}^{2}C_{Hs}^{2}(1-\rho_{c}^{2})>0.$$

$\Rightarrow {\left(1-k_{H}\right)}^{2}>0$, which is always true.

**(c) Comparison of** *MSE*_*opt*_[*q*_(*θ*,*u*,*η*,*ψ*)_] **with classical product median estimator ${\hat{H}}_{P}$**

Comparing (1.6) and (4.3), (*optimum*)*q*_(*θ*,*u*,*η*,*ψ*)_ will be more efficient than ${\hat{H}}_{P}$ if. That is $\Rightarrow {\left(1+k_{H}\right)}^{2}>0$, which is always true.

**(d) Comparison of** *MSE*_*opt*_[*q*_(*θ*,*u*,*η*,*ψ*)_] **with classical exponential ratio median estimator, ${\hat{H}}_{\exp R}$**

Comparing (1.7) and (4.3), (*optimum*)*q*_(*θ*,*u*,*η*,*ψ*)_ is more efficient than ${\hat{H}}_{\exp R}$ if $\delta H_{s}^{2}[C_{Hs}^{2}+\frac{C_{Hr}^{2}}{4}(1-4k_{H})]-\delta H_{s}^{2}C_{Hs}^{2}(1-\rho_{c}^{2})>0$ $\Rightarrow {\left(1-2k_{H}\right)}^{2}>0$, which is always true.

**(e) Comparison of** *MSE*_*opt*_[*q*_(*θ*,*u*,*η*,*ψ*)_] **with classical exponential product median estimator ${\hat{H}}_{\exp P}$**.

Comparing (1.8) and (4.3), (*optimum*)*q*_(*θ*,*u*,*η*,*ψ*)_ will be more efficient than ${\hat{H}}_{\exp P}$ if $\delta H_{s}^{2}[C_{Hs}^{2}+\frac{C_{Hr}^{2}}{4}(1+4k_{H})]-\delta H_{s}^{2}C_{Hs}^{2}(1-\rho_{c}^{2})>0$ $\Rightarrow {\left(1+2k_{H}\right)}^{2}>0$, which is always true.

**(f) Comparison of** *MSE*_*opt*_[*q*_(*θ*,*u*,*η*,*ψ*)_] **with classical chain ratio median estimator ${\hat{H}}_{CR}$**

Comparing (1.9) and (4.3), (*optimum*)*q*_(*θ*,*u*,*η*,*ψ*)_ will be more efficient than ${\hat{H}}_{CR}$. Hence,

$\Rightarrow (k_{H}^{2}+4k_{H}+4)>0$, which is always true.

**(g) Comparison of** *MSE*_*opt*_[*q*_(*θ*,*u*,*η*,*ψ*)_] **with that of [8] estimator $MSE({\hat{H}}_{pp}^{G})$**

For *MSE*_*opt*_[*q*_(*θ*,*u*,*η*,*ψ*)_]to be more efficient than $MSE({\hat{H}}_{pp}^{G})$ *MSE*_*opt*_[*q*_(*θ*,*u*,*η*,*ψ*)_]≤ $MSE({\hat{H}}_{pp}^{G})$

$$\Rightarrow \frac{\delta }{4}H_{S}^{2}C_{Hs}^{2}(1-\rho_{c}^{2})\leq \frac{H_{s}^{2}[\frac{\delta }{4}C_{Hs}^{2}(1-\rho_{c}^{2})-\frac{\delta^{2}}{64}C_{Hr}^{4}-\frac{\delta^{2}}{16}C_{Hs}^{2}C_{Hr}^{2}(1-\rho_{c}^{2})]}{1+\frac{\delta }{4}C_{Hs}^{2}(1-\rho_{c}^{2})}$$

$$\Rightarrow C_{Hs}^{2}(1-\rho_{c}^{2})\leq \frac{H_{s}^{2}[C_{Hs}^{2}(1-\rho_{c}^{2})-\frac{\delta }{16}C_{Hr}^{4}-\frac{\delta }{4}C_{Hs}^{2}C_{Hr}^{2}(1-\rho_{c}^{2})]}{1+\frac{\delta }{4}C_{Hs}^{2}(1-\rho_{c}^{2})}$$

$$\Rightarrow (1-\rho_{c}^{2})\leq \frac{\left[\frac{\left(1-\rho_{c}^{2}\right)}{4}-\frac{\delta C_{Hr}^{4}}{16C_{Hs}^{2}}-\frac{\delta }{4}C_{Hr}^{2}(1-\rho_{c}^{2})\right]}{1+\frac{\delta }{4}C_{Hs}^{2}(1-\rho_{c}^{2})}$$

$$\Rightarrow (1-\rho_{c}^{2})\geq \frac{4[\left(1-\rho_{c}^{2})-\frac{\delta C_{Hr}^{4}}{16C_{Hs}^{2}}-\frac{\delta }{4}C_{Hr}^{2}(1-\rho_{c}^{2}\right)]}{1+\frac{\delta }{4}C_{Hs}^{2}(1-\rho_{c}^{2})}$$

Since $1+\frac{\delta }{4}C_{Hs}^{2}(1-\rho_{c}^{2})>0$, $\frac{\delta C_{Hr}^{4}}{16C_{Hs}^{2}}>0$ and $\frac{\delta }{4}C_{Hr}^{2}(1-\rho_{c}^{2})>0$

Hence [8] estimator is more efficient than the proposed estimator.

**(h) Comparison of** *MSE*_*opt*_[*q*_(*θ*,*u*,*η*,*ψ*)_] **with that of [9] estimators**

For the proposed estimator to be more efficient than Irfan et al. (2021) estimator

*MSE*_*opt*_[*q*_(*θ*,*u*,*η*,*ψ*)_]≤*MSE*(*T*_*i*_(*d*))

*MSE*(*q*_(*θ*,*u*,*η*,*ψ*)_)≤*MSE*(*T*_*i*_(*d*))

$$\Rightarrow \frac{\delta H_{s}^{2}}{4}C_{Hs}^{2}(1-\rho_{c}^{2})\leq H_{s}^{2}[1-\frac{A_{2}A_{4}^{2}+A_{1}A_{5}^{2}-2A_{3}A_{4}A_{5}}{\left(A_{1}A_{2}-A_{3}^{2}\right)}]$$

$$\Rightarrow \frac{\delta }{4}C_{Hs}^{2}(1-\rho_{c}^{2})\leq [1-\frac{A_{2}A_{4}^{2}+A_{1}A_{5}^{2}-2A_{3}A_{4}A_{5}}{\left(A_{1}A_{2}-A_{3}^{2}\right)}]$$

From the works of [9] their proposed estimators are more efficient than [8] estimator. Therefore, [9] estimators are more efficient than proposed estimator.

**Remarks:** As it is stated that the estimators of [8, 9] are more efficient than the proposed estimator, however the practical applications of these estimators in the field become a bit cumbersome and sometimes almost infeasible because of the presence of the unknown parameters in the estimators. Even if the estimators of these unknown parameters could be determined, they may turn out to be biased. But our proposed classes of estimator do not have parameters that are unknown. The parameters found could be easily estimated from past or pilot surveys.

## 7. Empirical study

In this study, we have used data sets of four populations to reveal our general results and check the optimality performance of the AOE for recommended estimator *q*_(*θ*,*u*,*η*,*ψ*)_ over the fitted members of estimators mentioned in this study. The objective of using these four data sets is that, it is actually the situation where our suggested estimators perform better than existing ones as these data sets are not normal but follow skewed distributions. The results are presented in Tables 2–5, respectively for each population. The Percent Relative Efficiency (PRE) results are presented in Table 6. The populations are stated as:

**Table 2:** Bias and MSE values of members of the proposed class of estimators for population 1.

Estimators	Bias	Variance or MSE
${\hat{M}}_{y}$	0.00	565305.20
${\hat{M}}_{R}$	171.20	746571.60
${\hat{M}}_{P}$	83.55	1437668.00
${\hat{M}}_{\exp R}$	53.76	524234.70
${\hat{M}}_{\exp P}$	9.93	869783.10
${\hat{M}}_{CR}$	421.84	3363661.00
$\hat{M}({\hat{H}}_{pp}^{G})$	2.345	490067.81
** $\hat{M}(T_{i}(d))$ **	1.987	470987.56
$z_{\left(3,2,2195/5000,12/100\right)}$	-32.24	508641.50

**Table 3:** Bias and MSE values of members of the proposed class of estimators for population 2.

Estimators	Bias	Variance or MSE
${\hat{M}}_{y}$	0.00	28640.03
${\hat{M}}_{R}$	-14.28	23214.02
${\hat{M}}_{P}$	16.21	34752.58
${\hat{M}}_{\exp R}$	-7.38	25841.21
${\hat{M}}_{\exp P}$	7.86	31610.49
${\hat{M}}_{CR}$	34.34	41551.66
$\hat{M}({\hat{H}}_{pp}^{G})$	24.67	3907.67
** $\hat{M}(T_{i}(d))$ **	18.90	3709.45
** $z_{\left(7,6,5823/10000,9/11\right)}$ **	-307.02	4399.11

**Table 4:** Bias and MSE values of members of the proposed class of estimators for population 3.

Estimators	Bias	Variance or MSE
${\hat{M}}_{y}$	0.00	23.12
${\hat{M}}_{R}$	0.16	11.22
${\hat{M}}_{P}$	0.56	78.09
${\hat{M}}_{\exp R}$	-0.01	11.78
${\hat{M}}_{\exp P}$	0.19	45.22
${\hat{M}}_{CR}$	1.83	176.14
$\hat{M}({\hat{H}}_{pp}^{G})$	0.089	5.78
** $\hat{M}(T_{i}(d))$ **	0.076	4.76
** $z_{\left(-2,-3,80/3125,-29/56\right)}$ **	0.01	6.81

**Table 5:** Bias and MSE values of members of the proposed class of estimators for population 4.

Estimators	Bias	Variance or MSE
${\hat{M}}_{y}$	0.00	1.23
${\hat{M}}_{R}$	0.23	2.22
${\hat{M}}_{P}$	0.02	2.66
${\hat{M}}_{\exp R}$	0.08	1.42
${\hat{M}}_{\exp P}$	-0.02	1.64
${\hat{M}}_{CR}$	0.30	6.51
$\hat{M}({\hat{H}}_{pp}^{G})$	0.16	0.49
** $\hat{M}(T_{i}(d))$ **	0.15	0.31
** $z_{\left(5,4,1273/2500,42/79\right)}$ **	0.32	0.69

**Table 6:** PREs of the members of the proposed class of estimators.

Estimators	Population I	Population II	Population III	Population IV
${\hat{M}}_{y}$	100.00	100.00	100.00	100.00
${\hat{M}}_{R}$	75.72	123.37	205.99	55.17
${\hat{M}}_{P}$	39.32	82.41	29.60	46.17
${\hat{M}}_{\exp R}$	107.83	110.83	196.16	86.28
${\hat{M}}_{\exp P}$	64.99	90.60	51.12	74.87
${\hat{M}}_{CR}$	16.81	68.93	13.12	18.83
$\hat{M}({\hat{H}}_{pp}^{G})$	115.35	732.91	400.00	251.02
** $\hat{M}(T_{i}(d))$ **	120.02	772.08	485.71	396.77
***z***_**(*θ*,*u*,*η*,*ψ*)**_	111.14	651.04	339.67	178.18

### 7.1 Population I (see [17] for more details)

*M* = 69, *m* = 17, *ρ*_*c*_ = 0.3166, *H*_*s*_ = 2068, *C*_*Hs*_ = 3.45399, *C*_*Hr*_ = 3.33433.

From the above, we obtain

*t* = 0264, ⇒(1−*t*) = 0.736, *k*_*H*_ = 0.32796, $C_{Hs}^{2}=11.93005$, $C_{Hr}^{2}=11.1178$, and $H_{s}^{2}=4276624$. Now, setting *u* = 2 such that (*u*) satisfies, the condition in (3.3) implies *θ* = 3. Then, set *η* = 2195/5000 and *ψ* = 12/100 so that (2*η*+*ψ*) = (2*k*_*H*_/*u*), *k*_*H*_ = 0.32796, so that (4.1) is satisfied. Hence, putting (*θ*,*u*,*η*,*ψ*) = (3,2,2195/5000,12/100) in (3.2), for population I, the AOE for the suggested class of estimators for the population median *H*_*s*_ is obtained by

q(3,2,2195/5000,12/100)=H^s{3−2(H^rHr)4391000exp[3(H^r−Hr)25(H^r+Hr)]} (5.1)

with its bias and MSE to the first degree of approximation, respectively, as (Table 2)

$$Bias[q_{\left(3,2,2195/5000,12/100\right)}]=\frac{(1-t){\hat{H}}_{s}C_{Hr}^{2}}{m}(\frac{249}{1000}-\frac{429}{500}k_{H}),$$ (5.2)

$$MSE[q_{\left(3,2,2195/5000,12/100\right)}]=\frac{(1-t){\hat{H}}_{s}}{m}[C_{Hs}^{2}+\frac{499}{2000}C_{Hr}^{2}(3\frac{124}{125}-8k_{H})].$$ (5.3)

### 7.2 Population II (see [18] for more details)

*M* = 340, *m* = 150, *ρ*_*c*_ = 0.92, *H*_*s*_ = 178, *C*_*Hs*_ = 31.17635, *C*_*Hr*_ = 3.41315.

From the above, we obtain

*t* = 0.4412, ⇒(1−*t*) = 0.5588, *k*_*H*_ = 8.40345, $C_{Hs}^{2}=917.965$, $C_{Hr}^{2}=11.6496$, and $H_{s}^{2}=31684$. In population II, setting *u* = 6 such that (*u*) satisfies, the condition in (3.3) implies *θ* = 7. Then, set *η* = 5823/10000 and *ψ* = 9/11 so that (2*η*+*ψ*) = (2*k*_*H*_/*u*), *k*_*H*_ = 8.40345, so that (5.1) is satisfied. Hence, putting (*θ*,*u*,*η*,*ψ*) = (7,6,5823/10000,9/11) in (3.2), for population II, AOE for the suggested class of estimators for the population median *H*_*s*_ is obtained by

q(7,6,5823/10000,9/11)=H^s{7−6(H^rHr)582310000exp[9(H^r−Hr)11(H^r+Hr)]}, (5.4)

with its bias and MSE to the first degree of approximation, respectively, as (Table 3)

$$Bias[q_{\left(7,6,5823/10000,9/11\right)}]=\frac{(1-t){\hat{H}}_{s}C_{Hr}^{2}}{m}(\frac{64}{625}-\frac{189669}{40000}k_{H}),$$ (5.5)

$$MSE[q_{\left(7,6,5823/10000,9/11\right)}]=\frac{(1-t){\hat{H}}_{s}}{m}[C_{Hs}^{2}+\frac{99}{200}C_{Hr}^{2}(71\frac{95029}{250000}-24k_{H})].$$ (5.6)

### 7.3 Population III (see [19] for more details)

*M* = 396, *m* = 65, *ρ*_*c*_ = 0.84, *H*_*s*_ = 30, *C*_*Hs*_ = 2.82869, *C*_*Hr*_ = 2.73045.

From the above, we obtain *t* = 0.16415, ⇒(1−*t*) = 0.83585, *k*_*H*_ = 0.776112, $C_{Hs}^{2}=8.0015$, $C_{Hr}^{2}=9.74588$, and $H_{s}^{2}=900$. Now, in population III, setting *u* = −3 such that (*u*) satisfies, the condition in (3.3) implies *θ* = −2. Then, set *η* = 808/3125 and *ψ* = −29/56 so that (2*η*+*ψ*) = (2*k*_*H*_/*u*), *k*_*H*_ = 0.776112, so that (5.1) is satisfied. Hence, let (*θ*,*u*,*η*,*ψ*) = (−2,−3,808/3125,−29/56) in (3.2), for population III, AOE for the suggested class of estimators for the population median *H*_*s*_ is calculated by

q(−2,−3,808/3125,−29/56)=H^s{−2+3(H^rHr)8083125exp[−29(H^r−Hr)56(H^r+Hr)]} (5.7)

with its bias and MSE to the first degree of approximation, respectively, as in (Table 4)

$$Bias[q_{\left(-2,-3,808/3125,-29/56\right)}]=\frac{(1-t){\hat{H}}_{s}C_{Hr}^{2}}{m}(\frac{221}{40000}-\frac{2211}{2000000}k_{H}),$$ (5.8)

$$MSE[q_{\left(-2,-3,808/3125,-29/56\right)}]=\frac{(1-t){\hat{H}}_{s}}{m}[C_{Hs}^{2}-\frac{737}{4000000}C_{Hr}^{2}(-\frac{6633}{1000000}+12k_{H})].$$ (5.9)

### 7.4 Population IV (see [20] for more details)

*M* = 67, *m* = 23, *ρ*_*c*_ = 0.6624, *H*_*s*_ = 4.8, *C*_*Hs*_ = 2.73045, *C*_*Hr*_ = 2.71592.

For these quantities, we get *t* = 0.3433, ⇒(1−*t*) = 0.6567, *k*_*H*_ = 0.08932, $C_{Hs}^{2}=7.4554$, $C_{Hr}^{2}=7.37622$, and $H_{s}^{2}=23.04$. Consider population IV by setting *u* = 4 such that (*u*) satisfies, the condition in (3.3) implies *θ* = 5. Then, set *η* = 1273/2500 and *ψ* = 42/79 so that (2*η*+*ψ*) = (2*k*_*H*_/*u*), *k*_*H*_ = 0.08932, and then (5.1) is satisfied. Hence, for (*θ*,*u*,*η*,*ψ*) = (5,4,1273/2500,42/79) in (3.2) for population IV, AOE for the suggested class of estimators for the population median *H*_*s*_ is obtained by

q(5,4,1273/2500,42/79)=H^s{5−4(H^rHr)12732500exp[42(H^r−Hr)79(H^r+Hr)]}, (5.10)

with its bias and MSE to the first degree of approximation, respectively, as (Table 5)

$$Bias[q_{\left(5,4,1273/2500,42/79\right)}]=\frac{(1-t){\hat{H}}_{s}C_{Hr}^{2}}{m}(\frac{87}{250}-3\frac{100091}{1000000}k_{H}),$$ (5.11)

$$MSE[q_{\left(5,4,1273/2500,42/79\right)}]=\frac{(1-t){\hat{H}}_{s}}{m}[C_{Hs}^{2}-\frac{310009}{800000}C_{Hr}^{2}(24\frac{10009}{12500}-16k_{H})].$$ (5.12)

Next, we consider how much gain in percent relative efficiency over the fitted members of estimators of the recommended estimator. Using Formulae (5.13), the results are given in Table 6.


$$PRE(.,{\hat{M}}_{y})=\frac{Var({\hat{M}}_{y})}{Var(.)}\times 100\%$$ (5.13)

The AOE of the recommended class of estimators does have the same expression of mean square error as the unbiased linear regression estimator, which stands clearly from Eq (4.1) and is independent of scalars (*θ*,*u*,*η*,*ψ*).

While investigating whether AOE is more proficient than the fitted members of the suggested class of estimators captured in this present work, it is clearly revealed from Section 8 and Eq (4.1) that the AOE agrees with the work of [21]. The proposed ratio class of median estimator is preferred to the classical ratio median estimator. Under certain optimal conditions, the recommended class of ratio-cum-median estimator is an alternative estimator to the unbiased linear regression estimator, which confirms the results obtained by [22–24].

While adopting the suggested class of ratio-cum-median estimator on the data, we have observed that AOE only depends on the value of (*k*_*H*_) and the fixed value of the scalar (*u*). Although there is no fixed or global optimum estimator that has been proved numerically, the achievement in our recommended class of ratio-cum-median estimators was noticeable. Precisely, using the data of four populations, the AOEs were $z_{\left(3,2,2195/5000,12/100\right)}$, $z_{\left(7,6,5823/10000,9/11\right)}$, $z_{\left(-2,-3,808/3125,-29/56\right)}$ and $z_{\left(5,4,1273/2500,42/79\right)}$ for the population I, II, III, and IV, respectively. Whenever the optimality conditions are satisfied, the AOE performs much better than the other members of the proposed family of estimators. The optimal results are not always obtained from the existing estimators. However, our AOE will always give optimal results since the optimality conditions are dependent on values of (*k*_*H*_) of the population under consideration that are revealed from Tables 2–5. Furthermore, from Table 6, we reveal that there is a greater gain in the AOE optimal estimator’s efficiency for population median for all the four populations under consideration in the study. Hence, some of our proposed members of the class of median estimators are preferred to the sample median estimator and some existing estimators, which are members of the same class. In addition, we have shown that, with ancillary information, our proposed ratio- cum-median method will enhance precision in sample survey, and our findings agree with the same findings of previous researchers [25–28].

On the other hand, it is also observed that the [8, 9] estimators are more efficient than our proposed class of estimators, even at optimality conditions. This is indicated as the Mean squared errors of these estimators are less than the proposed class of estimators in the four populations. The increase in efficiency of these estimators is due to the presence of many unknown parameter estimates, since it is a well-known fact that MSE is a function of the number of parameters present. Hence, the proposed class of median estimators outcompete [8, 9] estimators, in terms of their simplicity convenience in application.

## 8. Conclusions

In this paper, we have proposed an efficient class of ratio-cum-median estimators of the population median using simple random sampling without replacement. This study comes with the conclusion that once the optimality conditions given in Eq (2.3) and Eq (4.1) are fulfilled, the AOE of the recommended class of ratio-cum-median estimators performs much better than the existing members of the suggested estimators. The real data analysis also shows the superiority of the proposed class of estimator compared to other class members. Based on our analyses and findings, we strongly recommend using the AOE of the proposed class of estimators in practical situations. For future work, the suggested class of ratio-cum-median estimators can be extended by considering it with a Stratified random sampling, systematic sampling and ranked set sampling and its modifications, see [29–32].
